# Risk of suicide after hospitalizations due to acute physical health conditions—a cohort study of the Norwegian population

**DOI:** 10.1186/s12916-024-03623-5

**Published:** 2024-09-16

**Authors:** Andreas Asheim, Sara Marie Nilsen, Ellen Rabben Svedahl, Silje L. Kaspersen, Ottar Bjerkeset, Imre Janszky, Johan Håkon Bjørngaard

**Affiliations:** 1 Regionalt Senter for Helsetjenesteutvikling, St. Olavs Hospital, Postboks 3250 Sluppen, Trondheim, N-7006 Norway; 2 NTNU, Institutt for Samfunnsmedisin Og Sykepleie, Postboks 8905, Trondheim, 7491 Norway; 3 Faculty of Nursing and Health Sciences, Nord University, PB 93, Levanger, 7601 Norway

**Keywords:** Suicide, Health Services Research, Emergency admissions, Somatic

## Abstract

**Background:**

It is well known that individuals recently discharged from psychiatric inpatient care face a high risk of suicide. Severe physical health conditions have also been linked to suicide risk. The risk of suicide following discharge from somatic hospitals is not known for individuals admitted due to acute physical health conditions.

**Methods:**

A Cohort study using data from the entire Norwegian population aged 12 years and older from 2008 to 2022 linked with information on health service use and cause of death. We used Cox regression with age as time axis to estimate sex-adjusted hazard ratios of suicide following discharge for ages 12 to 64 years and 65 years and older. We also performed analyses after excluding hospitalizations with indications of concurrent mental disorders, self-harm, or suicide attempts. To assess individual risk, we performed an adapted case-crossover analysis among discharged patients who died from suicide.

**Results:**

A total of 4 632,980 individuals aged 12 to 64 years and 1,469,265 individuals aged 65 years and older were included. Compared to unexposed individuals at similar ages, we found an increased risk of suicide in the first 4 weeks after discharge, with a hazard ratio (HR) of 7.0 (95% confidence interval (CI) 5.9 to 8.3) among those aged 12 to 64 years and 6.8 (95% CI 5.4 to 8.6) among those 65 years and older. In the younger age group, the risk was attenuated, with a HR of 2.4 (95% CI 1.7 to 3.2) after excluding hospitalizations with indications of concurrent mental disorders, self-harm, or suicide attempts. The corresponding HR was 4.8 (95% CI 3.5 to 6.4) among those 65 years and older, declining to 1.9, (1.2 to 3.1) in weeks 5 to 8 and 1.2 (0.7 to 2.2) in weeks 21 to 24. The case-crossover analysis confirmed that individuals 65 years and older were particularly vulnerable.

**Conclusions:**

The heightened risk of suicide following discharge from acute somatic hospitalization, even in the absence of concurrent mental disorders, self-harm, or prior suicide attempts, underscores the critical need for comprehensive mental health and existential support for patients post-discharge.

**Supplementary Information:**

The online version contains supplementary material available at 10.1186/s12916-024-03623-5.

## Background

Suicide mortality represents a major public health concern. Nearly 800,000 people worldwide die by intentional self-directed injury each year, about one person every 30 s [1]. In Norway, with a population of 5.4 million in 2021, 658 people died by suicide. Men account for about two out of every three cases [2]. While the relationship between risk and protective factors contributing to suicide is complex, and the success of long-standing suicide prevention efforts remains contested, recent evidence provides a more hopeful perspective [3].

It is well known that individuals recently discharged from a psychiatric hospital face a substantially increased risk of suicide [4, 5]. Mental and physical health are closely intertwined, and people diagnosed with severe physical health conditions—such as cardiovascular diseases, low-survival cancers, chronic obstructive pulmonary disease, chronic ischemic heart disease, or degenerative neurological diseases—have an increased risk of dying in suicide [6–12]. Psychological reactions to severe stress, such as post-traumatic stress disorder and adjustment disorders, are common after acute physical health events, like infections, accidents, and cardiovascular disease [13, 14]. There is evidence highlighting the importance of suicide risk assessment in healthcare settings outside of psychiatric care, indicating that specific somatic disorders should be targeted [15, 16]. The potential suicide risk after discharge from hospitalization for physical health conditions is likely to be particularly high in patients with a psychiatric history [17]. Additionally, the potentially vulnerable period following hospitalization for acute physical conditions is less studied and may often be overlooked within healthcare services.

There are several reasons why the post-discharge period may represent a vulnerable period. Many patients experience fluctuating emotional states after an acute somatic episode, for example when faced with a poor prognosis or having experienced a sudden loss of function [9, 10, 12]. Access to lethal means is a widely recognized contributing factor to suicide risk [18]. The availability of potentially lethal medications, which are commonly prescribed upon discharge, might elevate the risk of self-harm and suicide. Also, some physical health conditions, such as cancer [19] or cardiovascular diseases [6–10], or certain medications [20], can themselves trigger mood disorders via inflammatory pathophysiologic mechanisms [21]. A well-designed discharge process involves several key elements to ensure continuity of care and minimize the risk of adverse events. This process could be negatively influenced by strained hospital resources, potentially leading to expedited discharge and insufficient patient information, limited access to, and communication with, post-discharge support services, and increased reliance on primary health care [22]. In turn, this may impact the continuity and quality of patient care [22–24].

This study aims to comprehensively assess the risk of death by suicide after discharge from hospitalizations due to acute physical health conditions. We used a Cox survival model following the whole population while comparing risk among individuals who were recently discharged with those who were not. In a secondary analysis, we assessed a change in individual risk after a discharge with a self-controlled design.

## Methods

### Setting

Norway’s healthcare system provides universal access to medical services. Patients in urgent need of acute health care are usually referred to hospitals by primary care physicians [25], or they are picked up by the ambulance, and emergency department physicians determine if hospitalization is necessary. In cases of concurrent mental and physical illnesses, the urgency of their conditions determines the appropriate facility for initial treatment. For example, a patient with a suicide attempt with severe physical injury or intoxication will be admitted to a somatic hospital primarily and will at the time of discharge be considered for follow-up by or admission to a psychiatric facility. Notably, such patients will most likely get a discharge primary of secondary diagnose related to mental health conditions International Statistical Classification of Diseases and Related Health Problems (ICD-10) [26] F00-F99 (Mental and behavioural disorders). Patients admitted for self-injuries will most likely also get discharge diagnoses from ICD10 chapters S10-S19 (Neck injuries), S60-S69 (Injuries to the wrist and hand), T00-T98 (Injury, poisoning, and certain other consequences of external causes), and V01-Y98 (External causes of morbidity). In the context of registry data, this distinction leads to admissions being recorded as somatic if the patients were admitted to a somatic hospital, even in the cases of concurrent mental and physical illnesses.

### Data

We utilized demographic data from the entire Norwegian population, linked to the Norwegian Patient Registry, the Norwegian Cause of Death Registry, and the Norwegian Municipal Patient Registry. These include information on somatic hospital admissions occurring between January 1, 2008, and December 31, 2021. Our study considered hospital admissions defined by criteria outlined in Hassani and colleagues [27], such that each hospitalization encompasses the patient’s hospital entry, transfers within or between hospitals, and concludes upon the patient’s discharge. Throughout the study period, each patient was assigned a unique and anonymous identification number, enabling us to connect patient information across multiple registries. Data on cause-specific mortality was readily available from the Norwegian Cause of Death Registry.

### Primary outcome

The primary outcome was death by suicide, identified within the cause of death registry through the ICD-10 codes X60-X84 as the immediate or underlying cause of death. Suicides documented in the Norwegian Cause of Death Registry have been verified as accurate [28].

To investigate to what extent an association could be driven by suicide due to medication overdose, we analysed suicides by poisoning, specifically, ICD-10 codes X60-69.

### Exposure

An individual was considered exposed in the period after discharge from an acute admission to a somatic hospital. To balance sufficient statistical power and capture the potentially most vulnerable time period, as outlined in a previously published analysis protocol [29], we chose to focus on the initial 4 weeks after discharge. Results are also presented at 4-week intervals up to 24 weeks after discharge.

Our analysis focused solely on the risk of suicide following discharge from somatic hospital wards. Patients admitted to somatic hospitals due to self-harm, or suicide attempts may be of substantially increased vulnerability in the period after leaving the hospital. To address this, we present analyses before and after excluding admissions with primary or secondary diagnoses related to mental health conditions ICD10-codes F00-F99 (Mental and behavioural disorders), S10-S19 (Neck injuries), S60-S69 (Injuries to the wrist and hand), T00-T98 (Injury, poisoning, and certain other consequences of external causes), and V01-Y98 (External causes of morbidity). We also present results for these categories separately.

### Study design and statistical analyses

We studied individuals aged 12 years and older, as suicides are very rare among those under 12 [2]. We analysed individuals in two groups: those aged 12 to 64, and those 65 and older. This separation was introduced due to the higher prevalence of comorbidities and age-specific diseases in the elderly [30] population. Individuals were followed from July 1st, 2008, the year they entered the age group or country, whichever occurred last. They were followed until December 31, 2021, date of death, emigration or, in the year they left the age group, whichever occurred first. To address the potential impact of multiple consecutive acute hospitalizations, we implemented a truncation procedure that excluded information from subsequent discharges occurring within 180 days of an initial discharge. This approach ensured that we focused on distinct episodes of hospitalization, avoiding the cumulative effects of sequences of acute hospitalizations.

The time following discharge from an acute hospitalization was treated as a time-varying covariate, initially designated as 0 until a discharge occurred (if it did), and then changed to 1 after the discharge. Individuals were followed up to a hospital admission occurred, then, by the truncation procedure, followed from discharge to the next admission or 180 days, whichever occurred first. After 180 days, follow-up as unexposed was resumed. An individual could therefore be followed as exposed multiple times, illustrated in Additional file 1: Figure S1. Cox regression was used to estimate hazard ratios of suicide following discharge. We used age as the time axis, and analyses were adjusted for sex.

### By ICD-10 chapters

We hypothesized that certain physical health conditions could carry especially high suicide risk and therefore presenting our results by major groups of disorders could enable a more targeted assessment. Subgroup analyses based on ICD-10 chapters were chosen because these groupings are widely used and easily recognized by clinicians.

### Additional analyses

#### Subgroup analyses

Separate analyses were conducted for females and males, and for individuals with different levels of education, categorized into primary, secondary and higher and according to hospital type (university hospital or not). We also performed separate analyses for admissions involving a surgical procedure as this could indicate pain issues and potential use of pain medications, and for discharges from hospital stays for particularly painful conditions, conditions involving inflammation, and conditions with sudden loss of function or prognosis. See Additional file 1: Table S1 for a complete list of diagnoses. We also did a separate analysis where we excluded admissions by individuals who recently (180 days prior) had contact with Mental Health Services. Finally, successful discharge planning requires coordination both within and outside the hospital. Hence, we assessed the effects of discharges on weekends, holidays, and the days preceding them. These discharge days might limit the planned discharge process in both the hospital and primary care settings [22, 31, 32].

#### Other potential causes of excess mortality

Due to a non-neglectable risk of misclassification of suicides, we also performed analyses including other potential causes of excess mortality in the weeks following discharge. *Death from medication overdoses* was identified through ICD-10 codes X40-49 (accidental poisoning) and F10-19 (mental and behavioural disorders due to psychoactive substance use).

*Deaths from accidents* were identified through ICD10-codes V01-V99 (transport accidents), Y10-Y43 (accidents with undetermined cause), and W0n-X59 (other external causes of accidental injury). Finally, we also identified *death from uncertain causes* through ICD-10 codes R96-R99 (ill-defined and unknown causes of mortality).

#### Alternative analytical design

Individuals who are at greater risk of suicide may also be more likely to experience acute hospitalizations due to physical health conditions. To address this problem, we employed a case-case-time-control design, proposed by Wang and colleagues [33], which is an adapted version of the case-crossover design. In this analysis, we compared an individual’s odds of a discharge having occurred 4 weeks prior to the outcome with the odds in preceding 4-week periods up to 1 year prior. Due to the within-person comparison, stable characteristics like education or life-style factors could not confound the association between discharge and suicide risk in these analyses. However, the case-crossover design [34] is susceptible to exposure trends due to seasonal variations and aging, since the event being confined to the end of the follow-up period [35]. The case-case-time-control design adjust for exposure trends using time trends from future cases as controls [33]. The approach is described in more detail in our online analysis protocol [29]. We present odds ratios along with 95% confidence intervals, estimated by conditional logistic regression as a fixed effect estimator.

### Ethical approval and transparency

The Regional Committees for Medical and Health Research Ethics has approved the study, 2016/2159/REK Midt. An analysis protocol was published August 18th, 2023, and we have shared code for data management and analyses [29].

## Results

In total, 4,632,980 individuals from 12 to 64 years of age and 1,469,265 individuals aged 65 years and older were included in the analysis (Table 1). This encompasses all Norwegian inhabitants in these age groups during the study period from 1 July 2008 to 31 December 2021. There were 6719 suicides in the period. Additional file 1: Table S2 shows suicide rates by age and sex. In total, we considered 4.7 million discharges from acute hospital stays. Mean length of stay was 4.6 days, and 4.0% of stays lasted zero days (data not shown). Within the initial 4-week period after discharge, 146 cases of suicide were observed among those 12 to 64 years and 78 among those 65 years and older. Within 24 weeks, the corresponding numbers were 547 and 193. Additional information on the number of incidents and exposure status is included in Additional file 1: Table S3.

**Table 1:** Descriptives

	12–64 years		65 and older	
Number of individuals included	4,632,980		1,469,265	
Women (%)	2,248,355	49%	782,653	53%
Men	2,384,625	51%	686,612	47%
Education (%)				
Primary	999,398	22%	453,433	31%
Secondary	1,621,416	35%	692,429	47%
Higher	1,497,137	32%	311,930	21%
Number of discharges	2,736,187		1,930,466	
After excluding discharges with indications of concurrent mental disorders, self-harm, or suicide attempts (%)	2,121,916	78%	1,414,827	73%
100 k person-years follow-up (%)				
Total	471		118	
As unexposed	458	97%	108	92%
As exposed, post-discharge	11	2%	7	6%
Truncated, post-discharge	2	0%	3	2%
Outcomes (rate per 100 k person-years)				
Suicides	5581	11.8	1138	9.7
Suicides by medication overdose	1146	2.4	230	2.0
Deaths from medication overdose	4472	9.5	1365	11.6
Deaths from accidents	7119	15.1	11,021	93.5
Deaths from uncertain causes	1488	3.2	9400	79.8

**Fig. 1:**
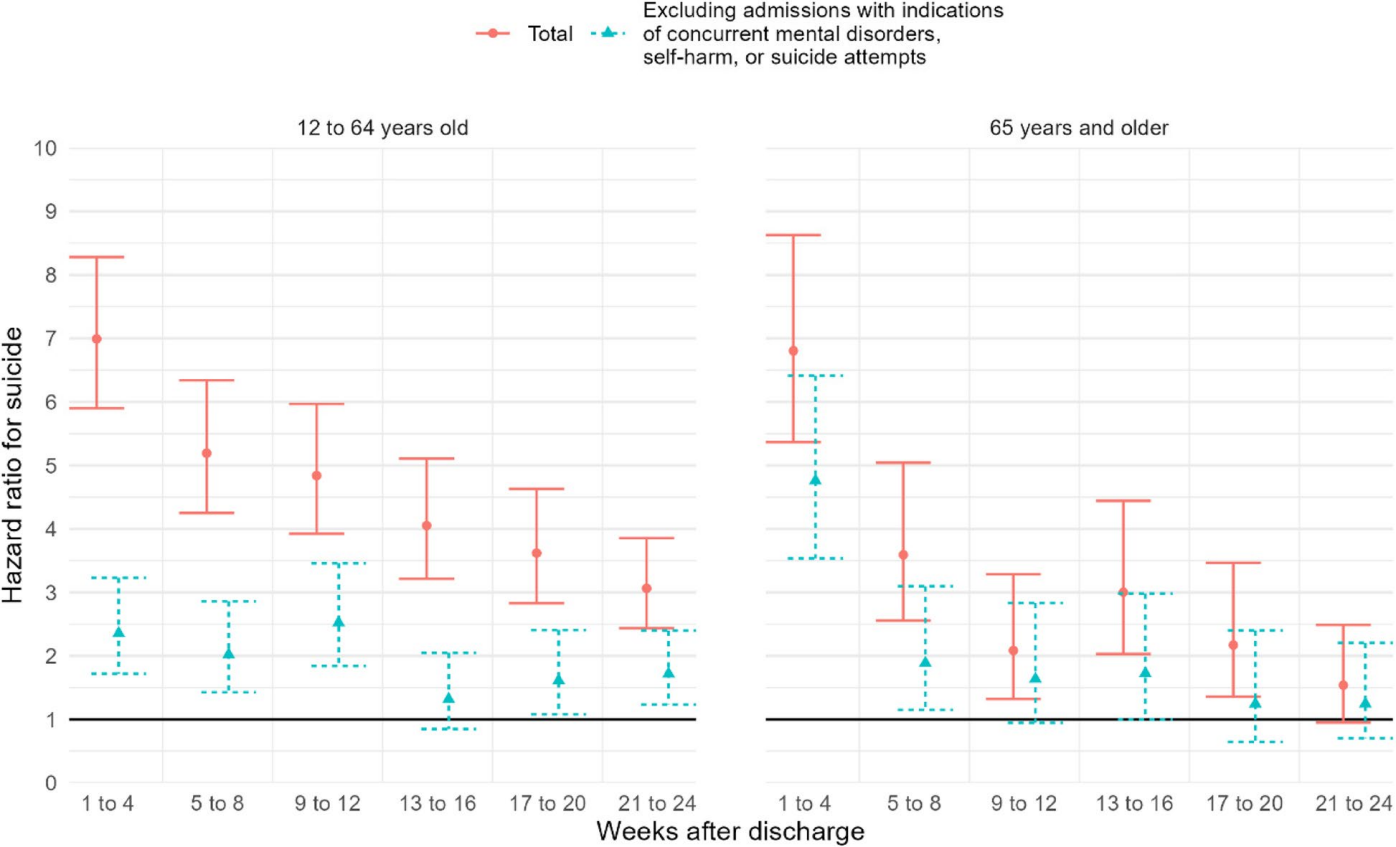
Hazard ratio for suicide per 4-week intervals up to 24 weeks after discharge from acute hospitalizations due to physical health conditions. Age was used as timescale. Adjusted for sex

Among individuals aged 12 to 64 years, the hazard ratio (HR) for suicide during the initial 4 weeks following discharge from acute somatic hospitalizations was 7.0 (95% confidence interval (CI) 5.9 to 8.3), as shown in Fig. 1 and Additional file 1: Figure S2. The corresponding HR was 6.8 (95% CI 5.4 to 8.6) among those 65 years and older. After excluding admissions with indications of concurrent mental disorders, self-harm, or suicide attempts, the HR was 2.4 (95% CI 1.7 to 3.2) among those 12 to 64 years old, and 4.8 (95% CI 3.5 to 6.4) among those 65 years and older.

In the youngest age group, after excluding admissions with indications of concurrent mental disorders, self-harm, or suicide attempts the HR remained at a relatively stable level up to 24 weeks after discharge (HR 1.6 95% CI 1.1 to 2.3 in weeks 21 to 24). Correspondingly, among individuals 65 years and older, the initial HR of 5.0 (95% CI 3.7 to 6.8) declined to 1.9 (95% CI 1.2 to 3.1) after 5 to 8 weeks and 1.2 (95% CI 0.7 to 2.2) after 21 to 24 weeks.

Analysing suicide due to poisoning as an outcome was compatible with the main results, as shown in Additional file 1: Figure S3.

Individuals diagnosed with a primary or secondary ICD-10 code indicating mental or behavioural disorders had an HR of 54 (95% CI 43 to 68) in the age groups 12 to 64 years old and an HR of 20 (95% CI 14 to 34) among those 65 years and older (Table 2). Admissions with indications of possible injuries from self-harm and external causes of morbidity were also followed by a substantially elevated risk of suicide during the initial 4 weeks following discharge.

**Table 2:** Hazard ratio for suicide within four weeks after discharge from acute hospitalizations due to physical health conditions, and by ICD-codes indicating concurrent mental disorders, self-harm, or suicide attempts

	12–64 years	65 and older
Hazard ratio for suicide 4 weeks after discharge	7.0 (5.9 to 8.3)	6.8 (5.4 to 8.6)
Discharges with primary or secondary diagnoses indicating concurrent mental disorders, self-harm, or suicide attempts:		
Mental and behavioural disorders (F00-F99)	54 (43 to 68)	20 (14 to 34)
Possible injuries from self-harm (S10-S19, S60-S69, T00-T98)	34 (26 to 44)	19 (11 to 33)
External causes of morbidity and mortality (V01-Y99)	17 (8.9 to 33)	8.8 (2.2 to 35)
Hazard ratio, when excluding admissions with indications of concurrent mental disorders, self-harm, or suicide attempts	2.4 (1.7 to 3.2)	4.8 (3.5 to 6.4)

Age was used as timescale. Adjusted for sex

### Analyses by ICD-10 chapters

Table 3 shows hazard ratios for suicide in the first 4 weeks after discharge, by primary diagnosis chapter, that is without exclusions based on secondary diagnoses. For both age groups, admissions with primary diagnoses from chapters F (mental disorders) and T (poisoning) showed the highest hazard ratios for suicide within the initial 4 weeks after discharge. For other ICD-10 chapters, primary diagnoses related to skin conditions (chapter L), injuries (chapter S), the endocrine system (chapter E) and the respiratory system (chapter J) showed the highest risk in the youngest age group, with hazard ratios around 10. In the oldest age group, diagnoses related to neoplasms (chapters C-D), skin conditions (chapter L), musculoskeletal system (chapter M) and symptoms or abnormal laboratory findings not elsewhere classified (chapter R) showed the highest risk, also with hazardous ratios around 10. Diseases of the circulatory system (chapter I) showed an HR of 2.9 (95% CI 1.4 to 5.7) among those 65 years and older.

**Table 3:** Hazard ratio for suicide within four weeks after discharge from hospitalizations due to acute physical health conditions, by primary diagnosis from ICD-chapters

	12–64 years	65 and older
ICD-10 chapter	*Hazard ratio* *(95% CI)*	*Hazard ratio* *(95% CI)*
I: Infections (A00-B99)	3.0 (0.74 to 12)	2.7 (0.38 to 19)
II: Neoplasms (C00-D48)	2.4 (0.33 to 17)	12 (5.8 to 25)
IV: Endocrine system (E00-E90)	9.2 (3.0 to 28)	5.3 (0.75 to 38)
IX: Circulatory system (I00-I99)	1.4 (0.46 to 4.4)	2.9 (1.4 to 5.7)
V: Mental and behavioural (F00-F99)	50 (35 to 71)	62 (33 to 120)
X: Respiratory system (J00-J99)	8.6 (4.5 to 16)	8.0 (4.3 to 15)
XI: Digestive system (K00-K93)	3.9 (2.0 to 7.9)	5.0 (2.1 to 12)
XII: Skin (L00-L99)	11 (3.7 to 35)	11 (1.5 to 78)
XIII: Musculoskeletal system (M00-M99)	1.9 (0.49 to 7.8)	9.3 (3.5 to 25)
XIV: Genitourinary system (N00-N99)	1.2 (0.17 to 8.5)	5.9 (2.2 to 16)
XIX—S: Injury (S00-S99)	9.3 (6.2 to 14)	3.2 (1.2 to 8.6)
XIX—T: Poisoning (T00-T98)	39 (28 to 54)	26 (13. to 52)
XVIII: Symptoms or laboratory findings (R00-R99)	5.5 (3.4 to 9.1)	9.4 (5.2 to 17)
VI: Nervous system (G00-G99)	2.9 (0.72 to 12)	Too few events

Age was used as timescale. Adjusted for sex

### Additional analyses

#### Sub-group analyses

In the youngest age group, the HR for suicide during the initial 4 weeks after discharge was 43 (95% CI 35 to 55) for individuals who had recent contact with psychiatric hospitals and 3.5 (95% CI 2.8 to 4.5) for those who did not (Additional file 1: Figure S4). A similar pattern was evident among those aged 65 years and older. We found comparable estimates as in the main analysis when analysing subgroups of hospital types, admission characteristics, the timing of discharge, the individual’s education level, and sex (Additional file 1: Figure S4). We analysed the risk per 4-week interval up to 24 weeks by sex and found comparable patterns (Additional file 1: Figure S5).

#### Other potential causes of excess mortality

Deaths from medication overdoses (Additional file 1: Figure S6), death from accidents (Additional file 1: Figure S7) and death from uncertain causes (Additional file 1: Figure S8) showed similar patterns as death from suicide.

#### Alternative analytical design

In the case-case-time-control analyses, 1260 individuals from 12 to 64 years of age and 420 individuals aged over 65 years were included. All included individuals had died from suicide after 2008 and experienced at least one acute admission to a somatic hospital during the study period. Compared to earlier 4-week periods, the odds of having had an acute hospitalization due to physical health conditions within the 4-week period before suicide was 1.42 times higher (95% CI 1.07 to 1.89) among individuals 12 to 64 years old, as shown in Table 4. Considering only discharges without indications of concurrent mental disorders, self-harm, or suicide attempts, the odds ratio was 1.05 (95% CI to 0.67 to 1.67). For individuals 65 years and older, the corresponding numbers were 3.21 (95% CI 1.87 to 5.50) when considering all acute hospitalization and 3.82 (95% CI 1.90 to 7.65) when excluding those with indications of concurrent mental disorders, self-harm, or suicide attempts. Results for secondary outcomes are shown in Additional file 1: Figure S9.

**Table 4:** Results from case-case-time-control analysis of suicide following discharges from acute hospitalizations for physical health conditions. Exposure trends were adjusted for by using time trends from future cases as controls

Odds ratio of suicide within 4 weeks of discharge (95% CI)	12 to 64 years	65 years or older
Discharges from any acute hospitalizations for physical health conditions	1.42 (1.07 to 1.89)	3.21 (1.87 to 5.50)
Excluding hospitalizations with indications of concurrent mental disorders, self-harm, or suicide attempts	1.05 (0.67 to 1.67)	3.82 (1.90 to 7.65)

## Discussion

This is the first study with a broad examination of the suicide risk after acute somatic hospitalization. Using data from 2008 to 2022, including the entire Norwegian population aged 12 years and older, we found a seven-fold increase in risk of suicide in the first 4 weeks following discharge after hospitalizations for acute physical health conditions. The increase in suicide risk was attenuated when we considered only admissions that did not involve concurrent mental disorders, self-harm, or suicide attempts. However, a five-fold increase in risk remained among individuals 65 years and older in this group.

### Connecting with previous research

There is evidence of a major increase in risk of suicide following discharge from psychiatric hospitals [4, 5]. In line with this, we found a 40-fold increase in suicide risk within the first 4 weeks after discharge in individuals aged 12 to 64, and a 20-fold increase in individuals aged 65 and older, among patients admitted to somatic hospitals with primary or secondary diagnoses indicating mental and behavioural disorders. Since admissions to somatic hospitals often involve cases related to mental health issues, self-harm, and suicide attempts, it is important to increase attention and care for these patients following both somatic and psychiatric hospitalizations.

Our main analyses focused on the change in risk between those who have just been discharged and the rest of the population, i.e., those who have never or not recently been discharged. Individuals with an increased risk of acute somatic hospitalizations may also have an overall increase in suicide risk which is not necessarily related to hospitalizations [36]. To address this potential source of confounding, we conducted a self-controlled case-case-time-control analysis [33] to examine the excess risk for suicide in relation to the first 4 weeks post-discharge from an acute hospital episode. This self-matching approach effectively controls for all stable characteristics of the patient, allowing us to assess individual excess risk. This design can also remove bias due to competing risk which might have not been negligible in our main analyses. In our Cox models, we censored individuals who died of other causes than suicide. However, individuals were at higher risk of mortality after a discharge, and therefore censoring after non-suicide deaths following a discharge was not uninformative. Additionally, the self-matching design helps pinpoint the particularly risky period just after discharge—a time when preventive action could be taken, as health services often have follow-ups after acute events. The case-crossover design is particularly prone to bias due to time trends and autocorrelation or carry-over effects between the comparison periods. We aimed to minimize the effects of time trends using the case-case-time-control controls design using the exposure trends from future cases to control for time trends. These analyses provided similar results to the main analyses, although the effect sizes were generally lower. We found that individuals aged 65 and older were particularly vulnerable in the 4-week post-discharge period. For younger patients, there was a modest increase in risk during this period. We found little evidence of increased suicide risk when considering only admissions without indications of concurrent mental disorders, self-harm, or suicide attempts. This largely corroborated our main findings, showing a gradual decline in risk after discharge in the younger population.

Experiencing acute illness can be deeply distressing, involving potential loss of function, dignity, and control, along with pain, uncertain prognosis, and the introduction of new medications. Many elderly patients with acute somatic health issues also struggle with despair and mental illness [37]. While it is well known that individuals diagnosed with severe physical health conditions have an increased risk of suicide [6–11], this study focuses on the specific period following discharge from an acute hospitalization. Acute hospitalization can sometimes indicate new cases of disease. However, it often represents a situation of rapid deterioration, necessitating immediate care, which may be both physically challenging and mentally traumatic. The discharge process is likely to be negatively influenced by strained hospital resources, leading to insufficient patient information, limited access to post-discharge support services, and increased reliance on primary health care [22]. As a result, many issues may not be addressed when patients leave the hospital. However, we did not find any difference in excess risk when considering discharges on weekends, holidays, and days preceding weekends and holidays, i.e., days that arguably have fewer resources and increased discharge pressure [24]. In Norway, patients discharged after acute somatic hospitalizations are screened for conditions like pressure sores, fall risks, and nutritional status [38]. Our results may strengthen the case for considering factors like loss of function, pain, mental well-being, and social support. Prioritizing mental health alongside physical well-being is essential for comprehensive care, especially for elderly patients facing complex health issues [39].

We conducted exploratory analyses of major disorder groups to identify potential targeted approaches. In both age groups, the post-discharge risk increased across all ICD-10 chapters. The particularly high risk in those with a main diagnosis related to mental and behavioural problems (chapter F in ICD-10) and poisoning (chapter T) could be interpreted as an increased risk after suicide attempts. Additionally, there was a substantially increased risk in those with a main diagnosis in the endocrine system (chapter E), respiratory system (chapter J), skin (chapter L), injury (chapter S), and symptoms or laboratory findings (chapter R). However, the confidence intervals were wide, so we should be cautious not to overinterpret the observed subgroup differences. Previous studies have indicated an increased risk after acute cardiovascular events, such as stroke and myocardial infarction [6–8, 10]. Our study indicates that this risk is not confined to specific somatic conditions but may be elevated following any acute hospitalization. It is worth noting that among those 65 years and older, the risk of suicide in the initial 4 weeks after discharge was of a similar or higher level for all groups of acute physical health conditions when compared to hospitalizations for circulatory diseases.

### Strengths and limitations

We used pre-defined analytical designs and approaches as described in a protocol that was locked and published online prior to running the analyses [29]. Further, following more than 4.6 million individuals aged 12 to 64 years and 1.4 million aged 65 years and older provided sufficient statistical power to estimate the risk of suicide in the initial 4 weeks following discharge. Nevertheless, suicide is a relatively rare event, and we lacked the statistical power to investigate effect measure modification in smaller groups of patients, particularly in younger age groups. Suicides documented in the Norwegian Cause of Death Registry have been verified as accurate [28]. Our supplementary analyses of other potential causes of excess mortality showed an increased risk in the 4-week period after discharge, with a gradual decline afterwards. We cannot rule out possible misclassification of suicides in these groups, and this finding may also indicate a general increase in mortality as a consequence of the underlying reason for admission. Hence, this should make our results conservative, as increased mortality could be a competing event for suicide.

Assessing the exact causes behind suicide deaths after discharge is a complex task and was not possible in the present work. Regardless of the specific reasons for the heightened risk of suicide, the period following discharge from the hospital presents an opportunity for preventive measures, as individuals often require ongoing medical attention in the weeks after leaving the hospital. In general, Norwegian hospital registry data are suggested to be complete and registration of main diagnoses to be fairly accurate [40]. However, we cannot rule out the potential misclassification of participants who may have had undiagnosed mental health disorders, as our register data might not capture all relevant diagnoses. Another limitation is that relevant information may have been lost by censoring additional acute admissions within 180 days, and the results could possibly not be applicable to suicide risk after series of hospitalizations.

## Conclusions

The increased risk of suicide following acute somatic hospitalization, even without recorded concurrent mental disorders, self-harm, or prior suicide attempts, highlights the need for comprehensive mental health and existential support for patients after discharge, particularly for the rapidly growing population of older patients dealing with complex health challenges.

## Supplementary Information

Additional file 1: Tables S1-S3 and Figures S1-S9. Figure S1: Follow-up and truncation procedure, Table S1: ICD-10 codes pain and sudden loss of function, Table S2: Outcomes per 100 000 person years, Table S3: Events and person-time, Figure S2: Hazard ratio for suicide up to 24 weeks after discharge, Figure S3: Hazard ratio for suicide by poisoning up to 24 weeks after discharge, Figure S4: Hazard ratio for suicide within initial four weeks by subgroups, Figure S5: Hazard ration for suicide up to 24 weeks after discharge by sex, Figure S6: Hazard ratio for death from medication overdoses up to 24 weeks after discharge, Figure S7: Hazard ratio for death from accidents up to 24 weeks after discharge, Figure S8: Hazard ratio for death from uncertain causes up to 24 weeks after discharge, Figure S9: Secondary outcomes in alternative analysis.

## Data Availability

The study data can be accessed from Norwegian registries at helsedata.no, as well as Statistics Norway. However, there are restrictions on availability. Each patient is assigned a unique, anonymous identification number during the observation period. Please note that these data were used under license for the current study and are not publicly accessible. An analysis protocol was published August 18th, 2023, and we have shared code for data management and analyses [29].

## Abbreviations

CI: Confidence interval
HR: Hazard ratio
ICD: International Statistical Classification of Diseases and Related Health Problems

## Data availability

The study data can be accessed from Norwegian registries at helsedata.no, as well as Statistics Norway. However, there are restrictions on availability. Each patient is assigned a unique, anonymous identification number during the observation period. Please note that these data were used under license for the current study and are not publicly accessible.
