# Correction: Risk of suicide after hospitalizations due to acute physical health conditions—a cohort study of the Norwegian population

**DOI:** 10.1186/s12916-024-03695-3

**Published:** 2024-10-17

**Authors:** Andreas Asheim, Sara Marie Nilsen, Ellen Rabben Svedahl, Silje L. Kaspersen, Ottar Bjerkeset, Imre Janszky, Johan Håkon Bjørngaard

**Affiliations:** 1 Regionalt Senter for Helsetjenesteutvikling, St. Olavs Hospital, Postboks 3250 Sluppen, N-7006 Trondheim, Norway; 2 NTNU, Institutt for Samfunnsmedisin Og Sykepleie, Postboks 8905, 7491 Trondheim, Norway; 3 Faculty of Nursing and Health Sciences, Nord University, PB 93, 7601 Levanger, Norway

**Correction** **:** **BMC Med 22, 396 (2024)**

**https://doi.org/10.1186/s12916-024-03623-5**

The original article [1] contained an error mistakenly carried forward by the production team which handled the article whereby third author, Ellen Rabben Svedahl was erroneously omitted as a co-first author; this omission has since been rectified.
