# Supplementary material for: Risk of suicide after hospitalizations due to acute physical health conditions—a cohort study of the Norwegian population
Source: BMC Med. 2024 Sep 16;22:396. doi: 10.1186/s12916-024-03623-5 (PMC11406799; doi:10.1186/s12916-024-03623-5)

Additional file 1

Figure S1: Illustration of follow-up and truncation procedure

Table S1: Lists of ICD-10 codes indication particularly painful conditions and conditions with sudden loss of function or prognosis

Table S2: Outcomes per 100 000 person years by age groups and sex

Table S3: Number of events and person-time in study

Figure S2: HR for suicide up to 24 weeks after discharge

Figure S3: HR for suicide by poisoning up to 24 weeks after discharge

Figure S4: HR for suicide within initial four weeks by subgroups

Figure S5: HR for suicide up to 24 weeks after discharge, by sex

Figure S6: HR for death from medication overdoses up to 24 weeks after discharge

Figure S7: HR for death from accidents up to 24 weeks after discharge

Figure S8: HR for death from uncertain causes up to 24 weeks after discharge

Figure S9: Secondary outcomes in alternative analysis

## Figure S1: Illustration of follow-up and truncation procedure

An example of a hypothetical individual who had four hospitalizations. Two of these occurred within 180 days of a previous hospitalization, and were not included in the analysis. As a result, the individual would be followed in five periods, alternating between unexposed and exposed.


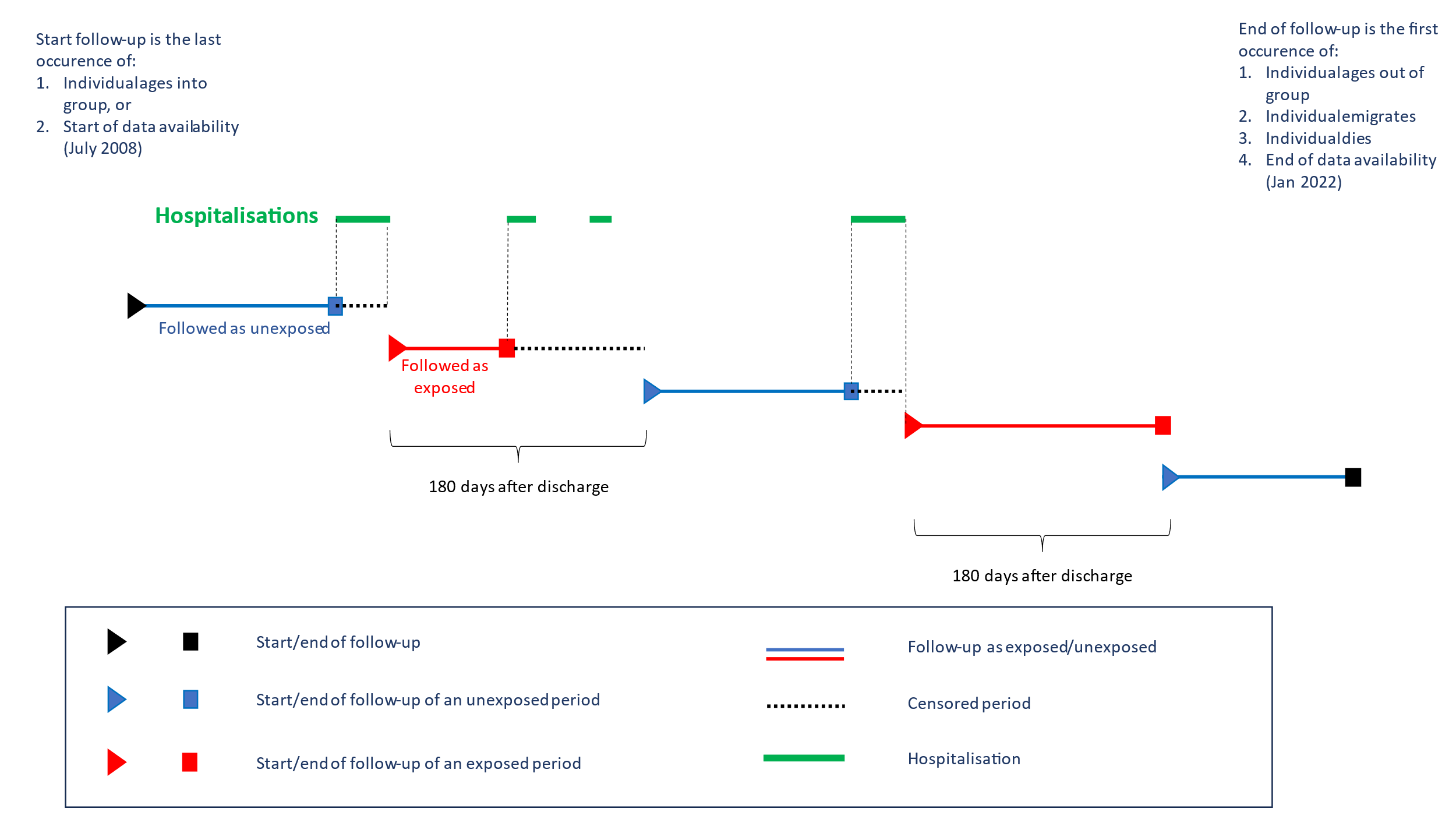


## Table S1: Lists of ICD-10 codes indication particularly painful conditions and conditions with sudden loss of function or prognosis

| **Particularly painful conditions** | | |  | **Conditions with sudden loss of function or prognosis** | | |
| --- | --- | --- | --- | --- | --- | --- |
|  | ICD-10 code | Description |  |  | ICD-10 code | Description |
|  | I60 | Subarachnoid haemorrhage |  |  | J09 | Influenza due to certain identified influenza virus |
|  | I61 | Intracerebral haemorrhage |  |  | J10 | Influenza due to other identified influenza virus |
|  | I62 | Other nontraumatic intracranial haemorrhage |  |  | J11 | Influenza, virus not identified |
|  | I63 | Cerebral infarction |  |  | J12 | Viral pneumonia, not elsewhere classified |
|  | I21 | Acute myocardial infarction |  |  | J13 | Pneumonia due to Streptococcus pneumoniae |
|  | I22 | Subsequent myocardial infarction |  |  | J14 | Pneumonia due to Haemophilus influenzae |
|  | I50 | Heart failure |  |  | J15 | Bacterial pneumonia, not elsewhere classified |
|  | J43 | Emphysema |  |  | J16 | Pneumonia due to other infectious organisms, not elsewhere classified |
|  | J44 | Other chronic obstructive pulmonary disease |  |  | J17 | Pneumonia in diseases classified elsewhere |
|  | T11.6 | Traumatic amputation of upper limb, level unspecified |  |  | J18 | Pneumonia, organism unspecified |
|  | T13.6 | Traumatic amputation of lower limb, level unspecified |  |  | J19 | Pneumonia, unspecified |
|  | T14.7 | Crushing injury and traumatic amputation of unspecified body region | |  | J20 | Acute bronchitis |
|  | Z89 | Refractory thrombocytopenia, malignant, metastatic site |  |  | J21 | Acute bronchiolitis |
|  | Z90 | Refractory thrombocytopenia, malignant, metastatic site |  |  | J22 | Unspecified acute lower respiratory infection |
|  | T31.1 | Burns involving 10-19% of body surface |  |  | N30 | Cystitis |
|  | T31.2 | Burns involving 20-29% of body surface |  |  | N10 | Acute tubulo-interstitial nephritis |
|  | T31.3 | Burns involving 30-39% of body surface |  |  | N39.0 | Urinary tract infection, site not specified |
|  | T31.4 | Burns involving 40-49% of body surface |  |  | A39 | Meningococcal infection |
|  | T31.5 | Burns involving 50-59% of body surface |  |  | A40 | Streptococcal sepsis |
|  | T31.7 | Burns involving 70-79% of body surface |  |  | A41 | Other sepsis |
|  | T31.8 | Burns involving 80-89% of body surface |  |  | A46 | Erysipelas |
|  | T31.9 | Burns involving 90% or more of body surface |  |  | A49 | Bacterial infection of unspecified site |
|  |  |  |  |  | A85 | Other viral encephalitis, not elsewhere classified |
|  |  |  |  |  | A86 | Unspecified viral encephalitis |
|  |  |  |  |  | A87 | Viral meningitis |
|  |  |  |  |  | M05 | Seropositive rheumatoid arthritis |

## Table S2: Outcomes per 100 000 person years by age groups and sex

Outcomes per 100 000 person years, summarized by age-group

|  |  | Suicide | |  | Suicide by poisoning | |  | Accidents | |  | Uncertain causes | |
| --- | --- | --- | --- | --- | --- | --- | --- | --- | --- | --- | --- | --- |
|  |  | Men | Women |  | Men | Women |  | Men | Women |  | Men | Women |
| (12,20] |  | 7.2 | 3.9 |  | 0.3 | 0.3 |  | 9.7 | 4.1 |  | 0.4 | 0.2 |
| (20,30] |  | 17.6 | 5.8 |  | 1.9 | 1.5 |  | 22.6 | 5.2 |  | 1.1 | 0.5 |
| (30,40] |  | 17.2 | 6.5 |  | 3.0 | 2.2 |  | 24.6 | 5.2 |  | 1.9 | 0.6 |
| (40,50] |  | 19.4 | 8.1 |  | 3.3 | 2.9 |  | 26.0 | 8.8 |  | 4.0 | 1.1 |
| (50,60] |  | 20.6 | 9.9 |  | 3.8 | 4.4 |  | 33.1 | 12.8 |  | 12.0 | 3.8 |
| (60,70] |  | 17.5 | 7.7 |  | 2.7 | 3.0 |  | 38.2 | 17.2 |  | 25.1 | 10.5 |
| (70,80] |  | 16.4 | 5.0 |  | 1.7 | 2.2 |  | 77 | 47 |  | 53 | 32 |
| (80,90] |  | 20.6 | 3.4 |  | 2.5 | 1.0 |  | 361 | 275 |  | 189 | 161 |
| >90 |  | 19.9 | 1.7 |  | 2.3 | 0.9 |  | 1265 | 995 |  | 543 | 585 |

## Table S3: Number of events and person-time in study

Summary of number of events in total, and within 4 and 24 weeks after discharge from acute hospitalizations due to physical health conditions, person years and incident rates.

|  |  | Ages 12-64 | | |  | 65 years and older | | |
| --- | --- | --- | --- | --- | --- | --- | --- | --- |
|  |  | Number of events | 10k person years | IR per 10k p.y. |  | Number of events | 10k person years | IR per 10k p.y. |
| **Suicides** | | 5 407 | 4 713 | 1.1 |  | 1 103 | 1 177 | 0.9 |
|  | Within 4 weeks of discharge | 140 | 21 | 6.7 |  | 71 | 15 | 4.8 |
|  | Excluding admissions with concurrent mental health issues | 31 | 16 | 1.9 |  | 41 | 11 | 3.8 |
|  | Within 24 weeks of discharge | 527 | 135 | 3.9 |  | 185 | 95 | 2.0 |
|  | Excluding admissions with concurrent mental health issues | 147 | 105 | 1.4 |  | 98 | 69 | 1.4 |
|  |  |  |  |  |  |  |  |  |
| **Suicides by poisoning** | | 1 106 | 4 713 | 0.2 |  | 224 | 1 177 | 0.2 |
|  | Within 4 weeks of discharge | 37 | 21 | 1.8 |  | 13 | 15 | 0.9 |
|  | Excluding admissions with concurrent mental health issues | <10 | 16 | <0.6 |  | <10 | 11 | <0.9 |
|  | Within 24 weeks of discharge | 140 | 135 | 1.0 |  | 41 | 95 | 0.4 |
|  | Excluding admissions with concurrent mental health issues | 46 | 105 | 0.4 |  | 18 | 69 | 0.3 |
|  |  |  |  |  |  |  |  |  |
| **Death from medication overdoses** | | 4 286 | 4 713 | 0.9 |  | 1 322 | 1 177 | 1.1 |
|  | Within 4 weeks of discharge | 137 | 21 | 6.5 |  | 79 | 15 | 5.4 |
|  | Excluding admissions with concurrent mental health issues | 28 | 16 | 1.7 |  | 24 | 11 | 2.2 |
|  | Within 24 weeks of discharge | 578 | 135 | 4.3 |  | 210 | 95 | 2.2 |
|  | Excluding admissions with concurrent mental health issues | 148 | 105 | 1.4 |  | 64 | 69 | 0.9 |
|  |  |  |  |  |  |  |  |  |
| **Death from accidents** | | 6 808 | 4 713 | 1.4 |  | 10 678 | 1 177 | 9.1 |
|  | Within 4 weeks of discharge | 148 | 21 | 7.1 |  | 2 780 | 15 | 188.7 |
|  | Excluding admissions with concurrent mental health issues | 44 | 16 | 2.7 |  | 154 | 11 | 14.3 |
|  | Within 24 weeks of discharge | 614 | 135 | 4.6 |  | 4 296 | 95 | 45.4 |
|  | Excluding admissions with concurrent mental health issues | 176 | 105 | 1.7 |  | 391 | 69 | 5.6 |
|  |  |  |  |  |  |  |  |  |
| **Death from uncertain causes** | | 1 404 | 4 713 | 0.3 |  | 9 025 | 1 177 | 7.7 |
|  | Within 4 weeks of discharge | 28 | 21 | 1.3 |  | 447 | 15 | 30.3 |
|  | Excluding admissions with concurrent mental health issues | 17 | 16 | 1.0 |  | 265 | 11 | 24.5 |
|  | Within 24 weeks of discharge | 113 | 135 | 0.8 |  | 1 363 | 95 | 14.4 |
|  | Excluding admissions with concurrent mental health issues | 69 | 105 | 0.7 |  | 786 | 69 | 11.3 |
| *IR: Incidence rate* | | | | | |  |  |  |

##

## Figure S2: HR for suicide up to 24 weeks after discharge

Hazard ratio for suicide up to 24 weeks after discharge from acute hospitalizations due to physical health conditions


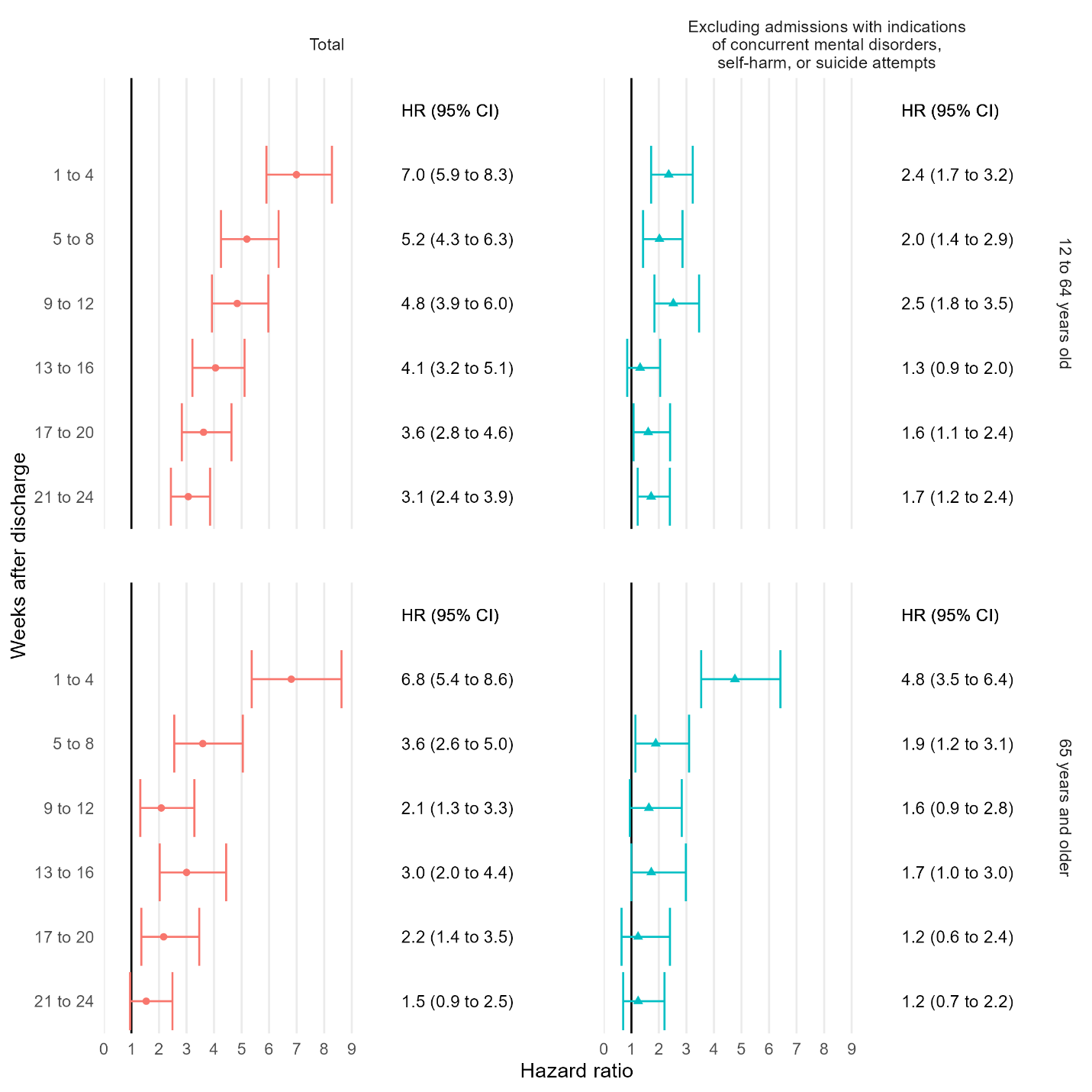


Age was used as the timescale. Adjusted for sex.

## Figure S3: HR for suicide by poisoning up to 24 weeks after discharge

Hazard ratio for suicide by poisoning per four-week intervals up to 24 weeks after discharge from acute hospitalizations


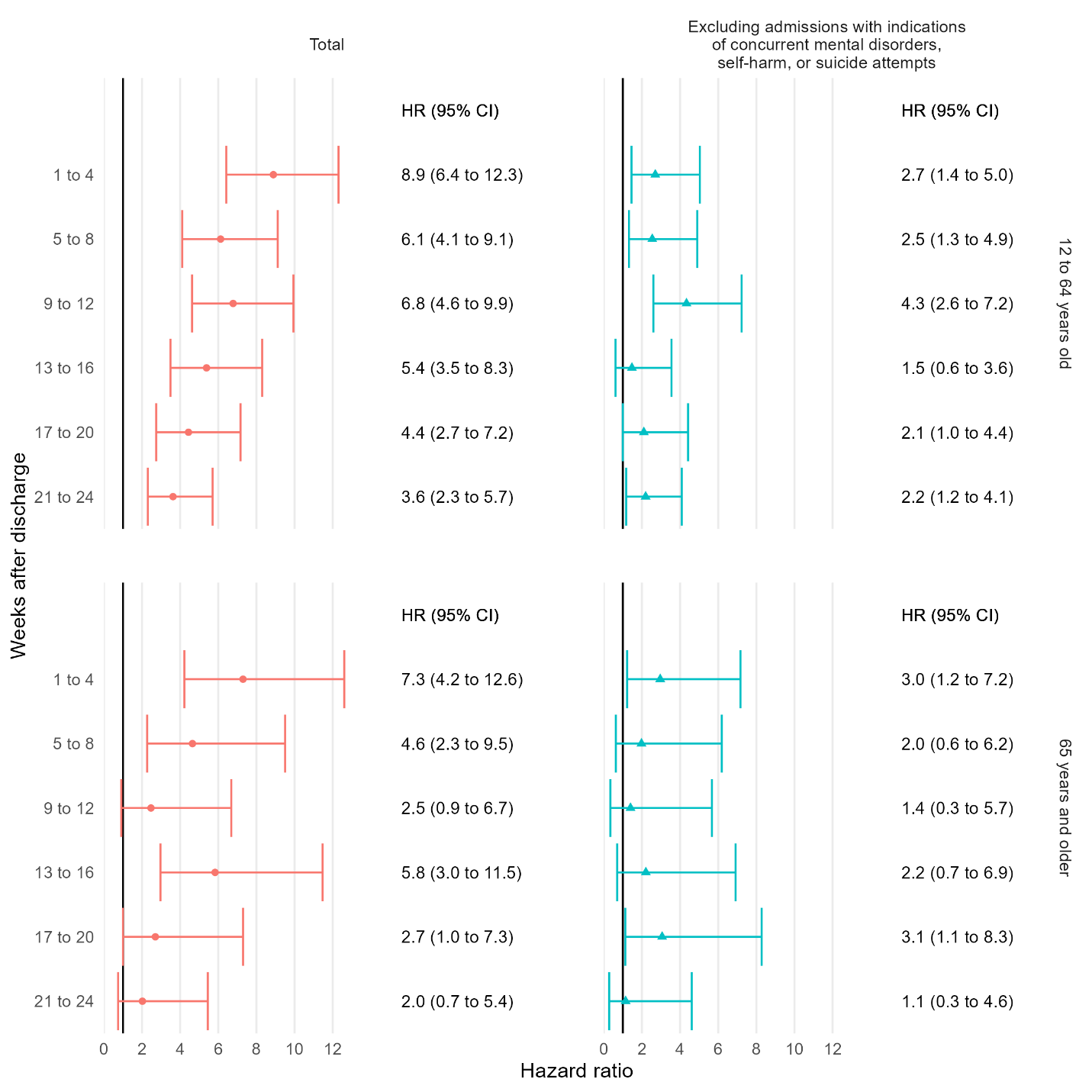


Age was used as the timescale. Adjusted for sex.

## Figure S4: HR for suicide within initial four weeks by subgroups

Hazard ratio for suicide within four weeks after discharge from acute hospitalizations due to physical health conditions by subgroups


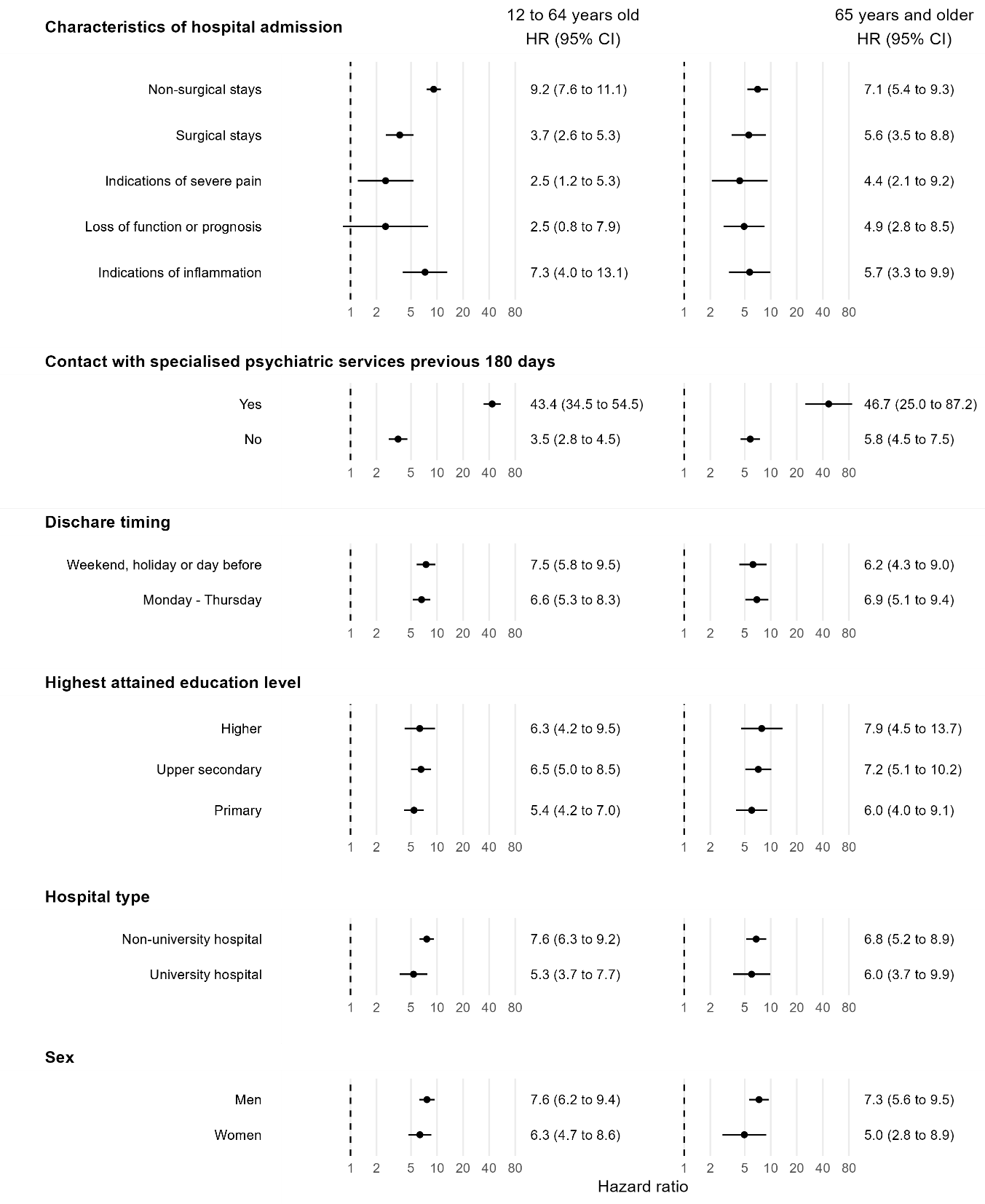


Age was used as the timescale. Adjusted for sex.

## Figure S5: HR for suicide up to 24 weeks after discharge, by sex

Hazard ratio for suicide up to 24 weeks after discharge from acute hospitalizations due to physical health conditions


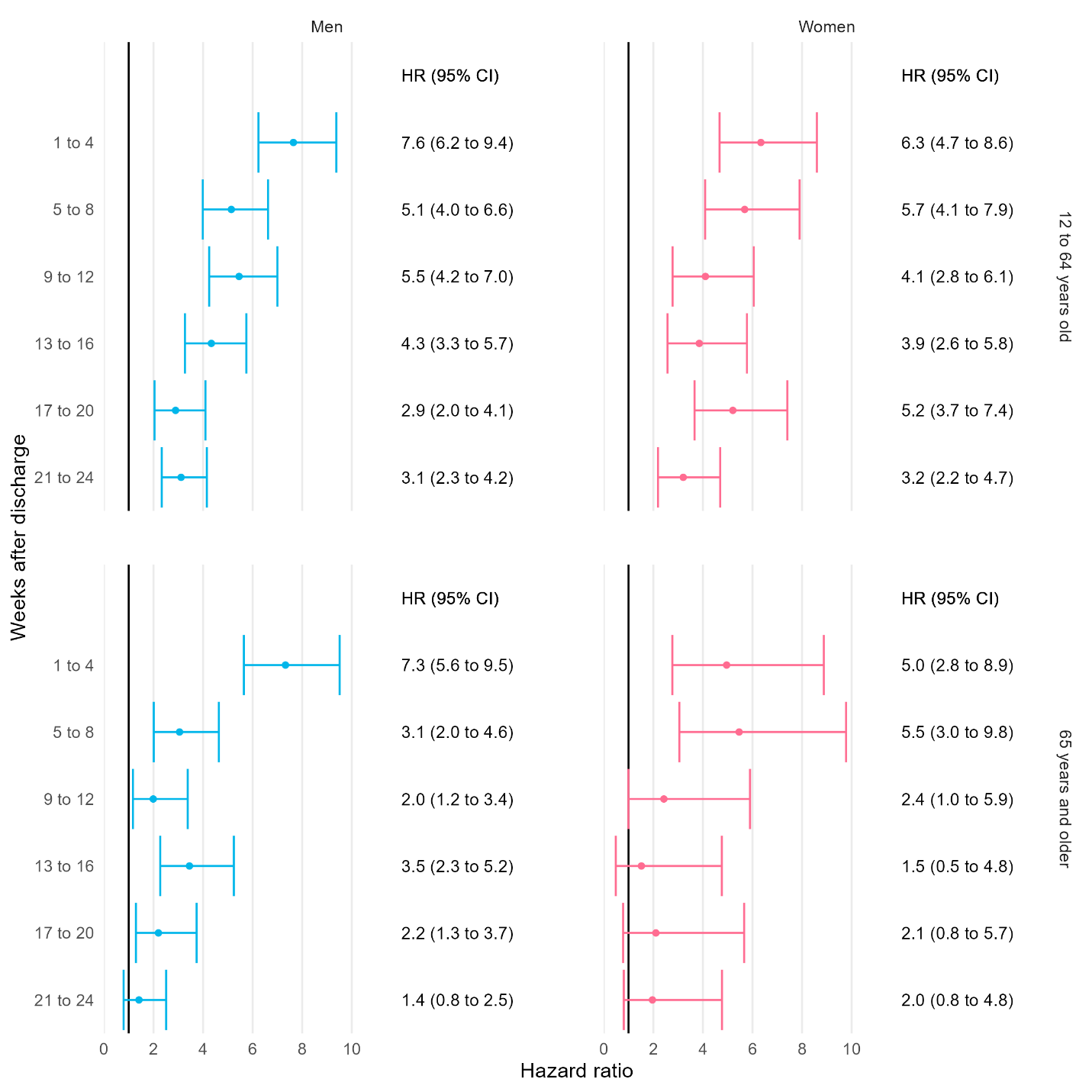


Age was used as the timescale.

## Figure S6: HR for death from medication overdoses up to 24 weeks after discharge

Hazard ratio for death from medication overdose per four-week intervals up to 24 weeks after discharge from acute hospitalizations


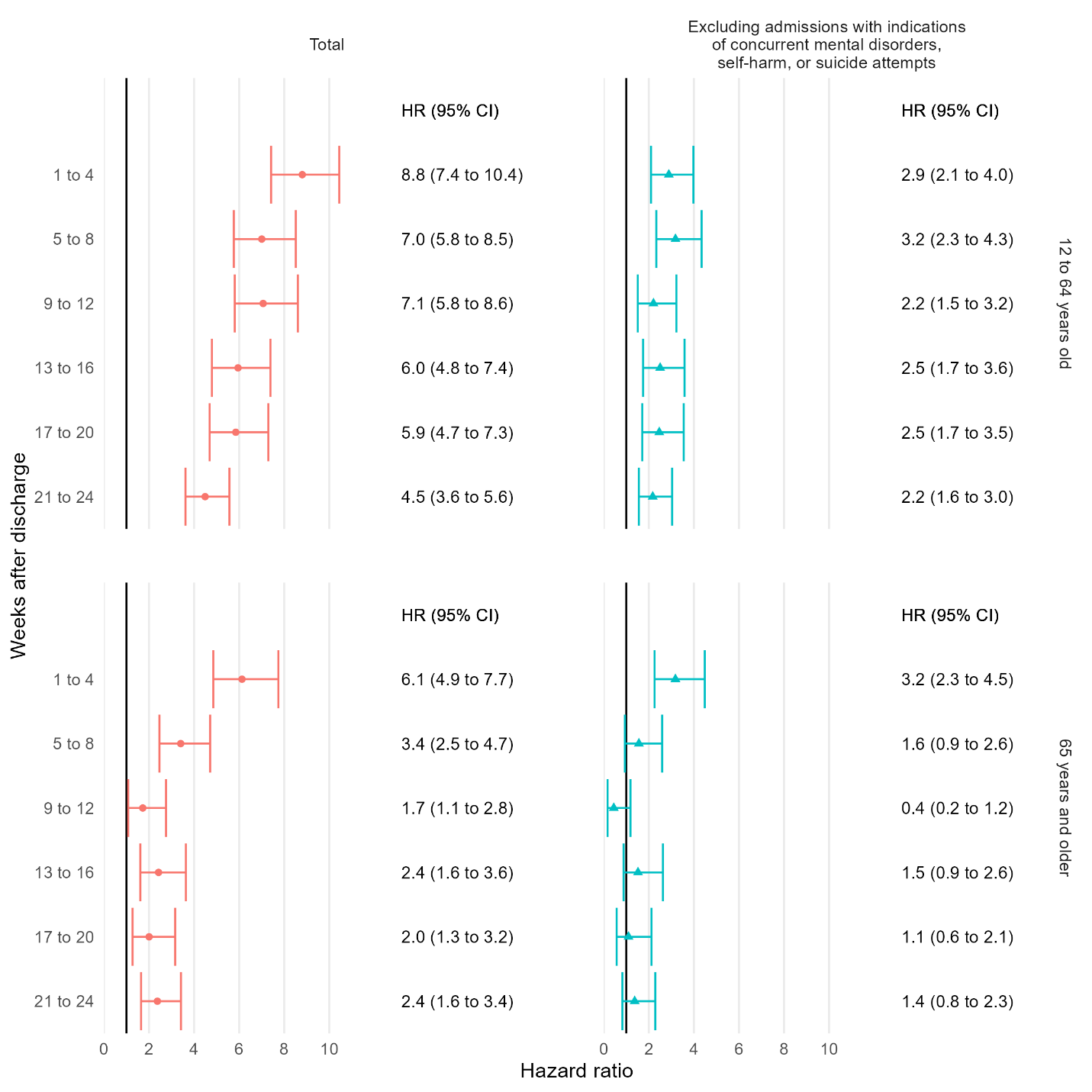


Age was used as the timescale. Adjusted for sex.

## Figure S7: HR for death from accidents up to 24 weeks after discharge

Hazard ratio for death from accidents per four-week intervals up to 24 weeks after discharge from acute hospitalizations


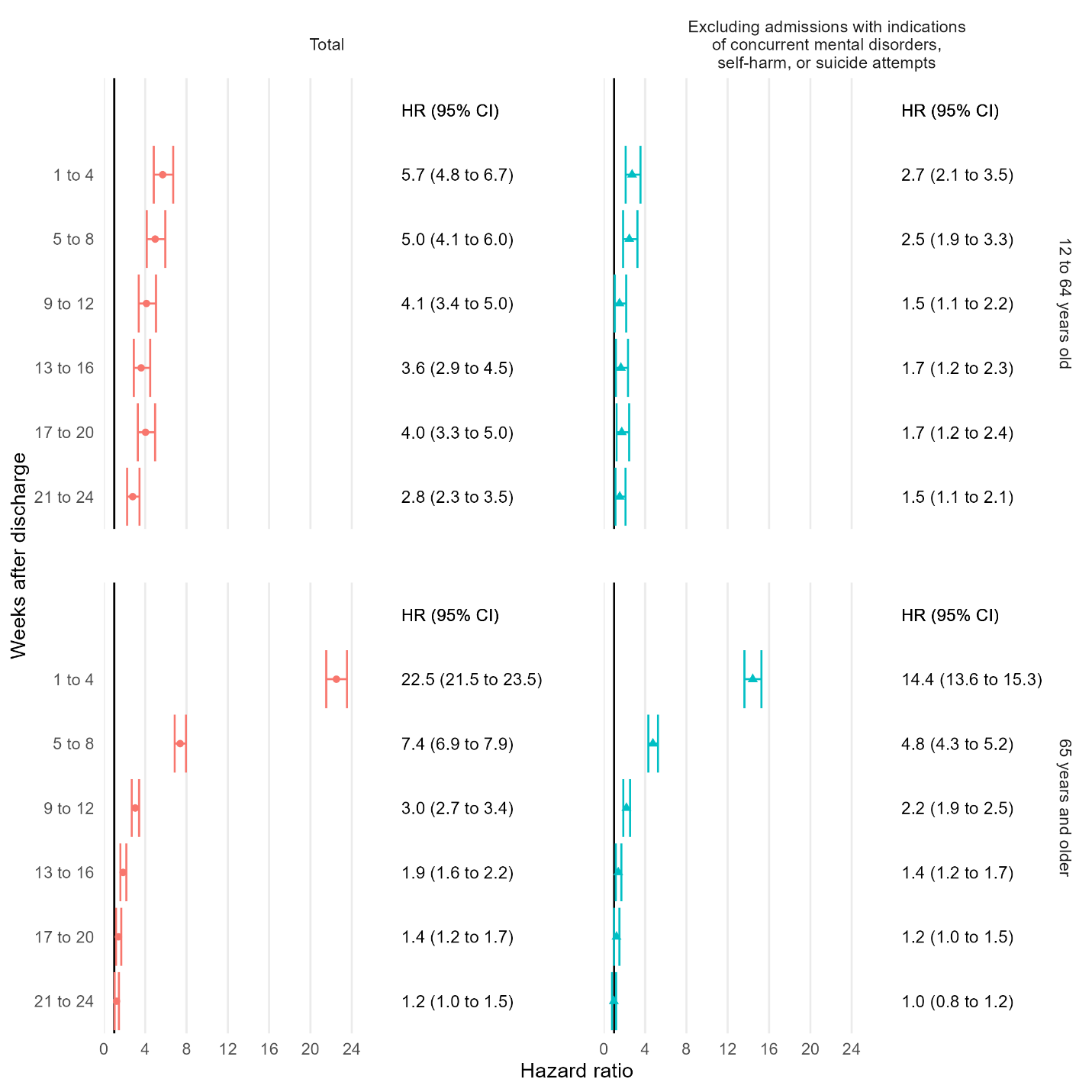


Age was used as the timescale. Adjusted for sex.

## Figure S8: HR for death from uncertain causes up to 24 weeks after discharge

Hazard ratio for death from uncertain causes accidents per four-week intervals up to 24 weeks after discharge from acute hospitalisations


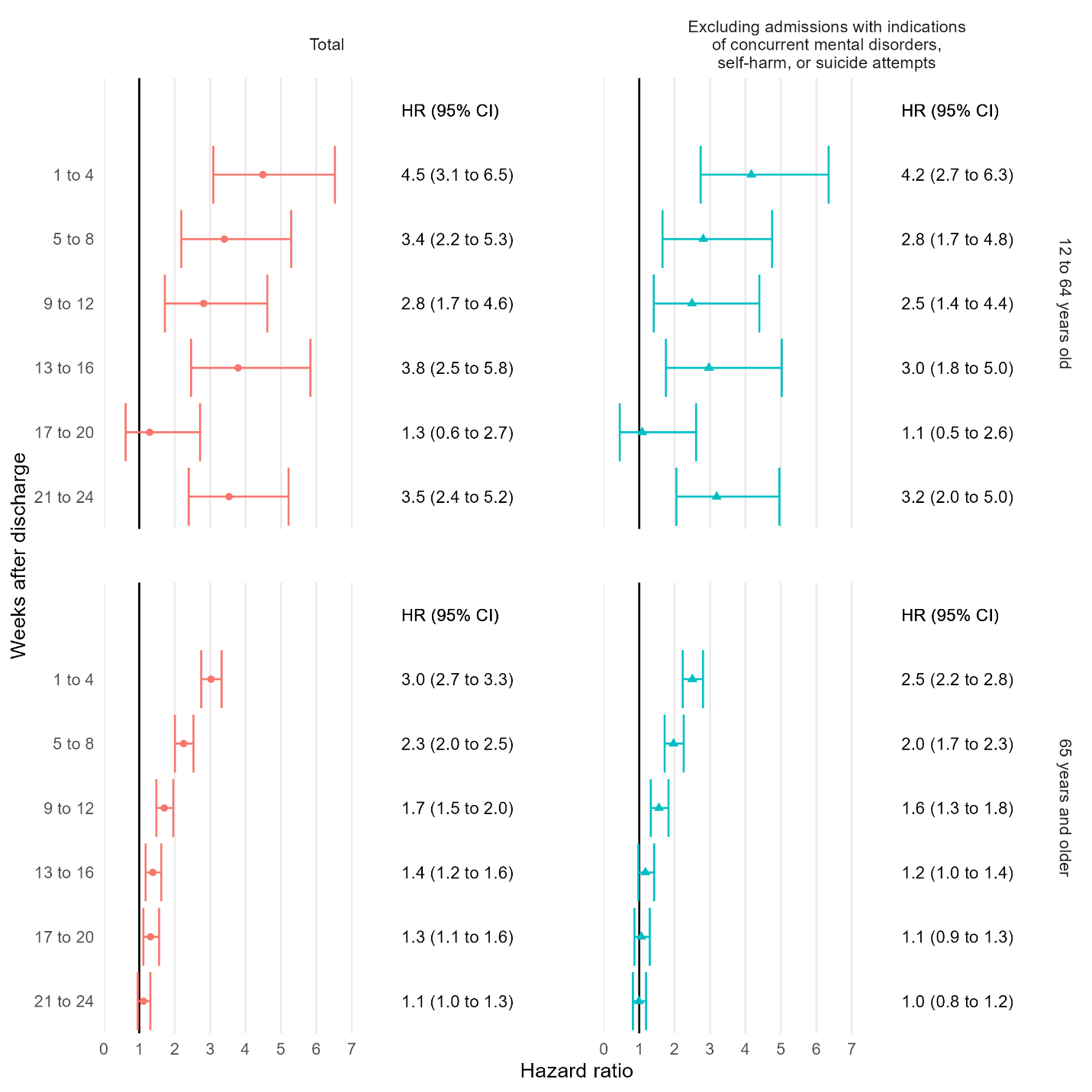


Age was used as the timescale. Adjusted for sex.

## Figure S9: Secondary outcomes in alternative analysis

Odds ratios for having been discharged four weeks prior to death, relative to other four-week periods up to one year prior for secondary outcomes. Time trends were adjusted for by using future cases as controls.


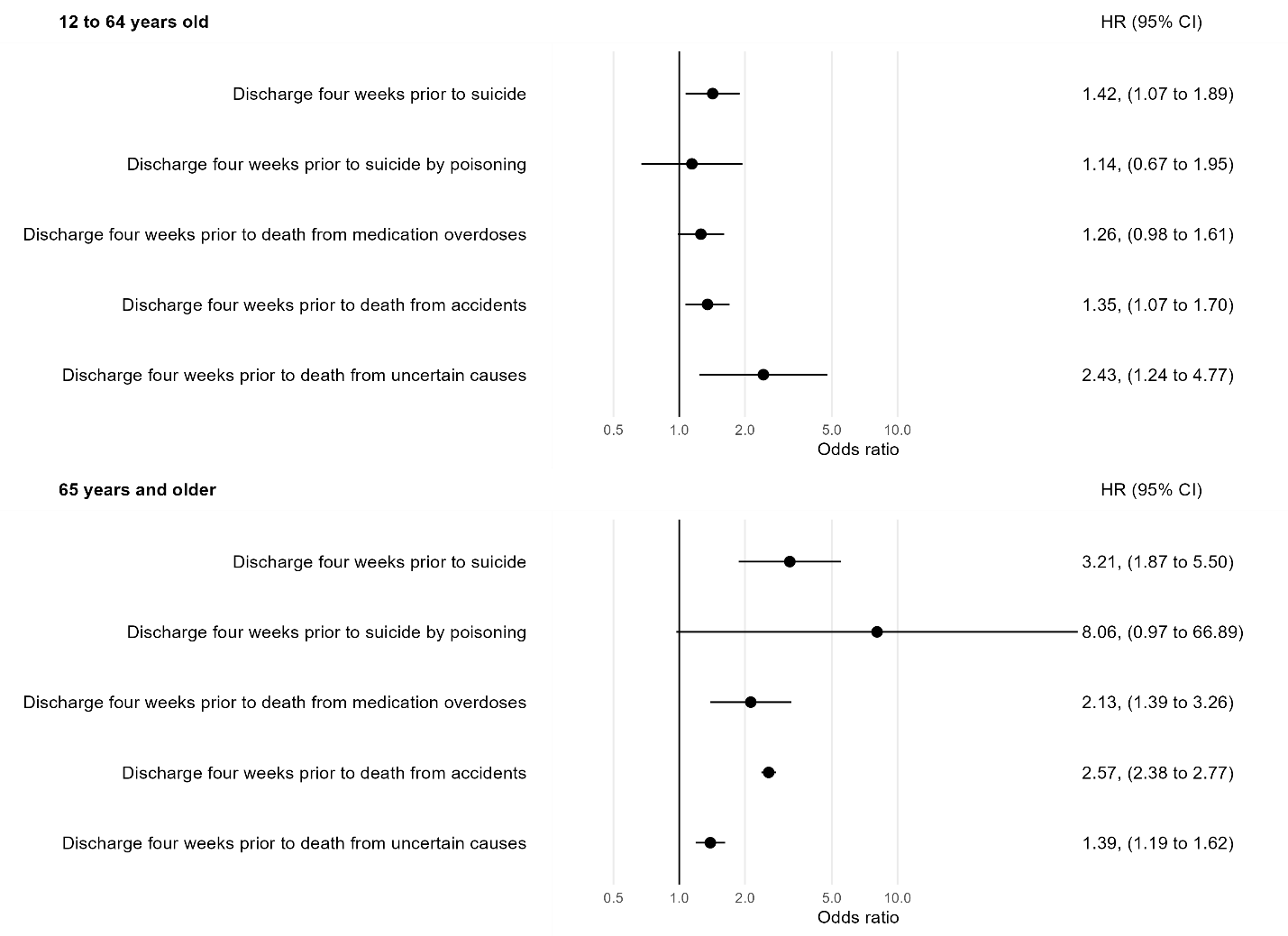

Supplement: Supplementary file 1 — Additional file 1: Tables S1-S3 and Figures S1-S9. Figure S1: Follow-up and truncation procedure, Table S1: ICD-10 codes pain and sudden loss of function, Table S2: Outcomes per 100 000 person years, Table S3: Events and person-time, Figure S2: Hazard ratio for suicide up to 24 weeks after discharge, Figure S3: Hazard ratio for suicide by poisoning up to 24 weeks after discharge, Figure S4: Hazard ratio for suicide within initial four weeks by subgroups, Figure S5: Hazard ration for suicide up to 24 weeks after discharge by sex, Figure S6: Hazard ratio for death from medication overdoses up to 24 weeks after discharge, Figure S7: Hazard ratio for death from accidents up to 24 weeks after discharge, Figure S8: Hazard ratio for death from uncertain causes up to 24 weeks after discharge, Figure S9: Secondary outcomes in alternative analysis. [file 12916_2024_3623_MOESM1_ESM.docx]
